# Percutaneous tibial nerve stimulation (PTNS) efficacy in the treatment of lower urinary tract dysfunctions: a systematic review

**DOI:** 10.1186/1471-2490-13-61

**Published:** 2013-11-25

**Authors:** Gabriele Gaziev, Luca Topazio, Valerio Iacovelli, Anastasios Asimakopoulos, Angelo Di Santo, Cosimo De Nunzio, Enrico Finazzi-Agrò

**Affiliations:** 1 School of Specialization in Urology, University of Rome Tor Vergata, Rome, Italy; 2 Department of Urology, Policlinico Casilino, Rome, Italy; 3 Department of Neuro-Urology, Fondazione S. Lucia, IRCCS, Rome, Italy; 4 Department of Urology, Sant'Andrea Hospital, Faculty of Health Sciences "La Sapienza" University of Rome, Rome, Italy; 5 Department of Urology, Policlinico Tor Vergata, Rome, Italy; 6 Department of Experimental Medicine and Surgery, University of Rome Tor Vergata, Rome, Italy

**Keywords:** PTNS, OAB, Lower urinary tract dysfunctions, Neurogenic bladder

## Abstract

**Background:**

Percutaneous Tibial Nerve Stimulation (PTNS) has been proposed for the treatment of overactive bladder syndrome (OAB), non-obstructive urinary retention (NOUR), neurogenic bladder, paediatric voiding dysfunction and chronic pelvic pain/painful bladder syndrome (CPP/PBS). Despite a number of publications produced in the last ten years, the role of PTNS in urinary tract dysfunctions remains unclear. A systematic review of the papers on PTNS has been performed with the aim to better clarify potentialities and limits of this technique in the treatment of OAB syndrome and in other above mentioned urological conditions.

**Methods:**

A literature search using MEDLINE and ISI web was performed. Search terms used were “tibial nerve” and each of the already mentioned conditions, with no time limits. An evaluation of level of evidence for each paper was performed.

**Results:**

PTNS was found to be effective in 37-100% of patients with OAB, in 41-100% of patients with NOUR and in up to 100% of patients with CPP/PBS, children with OAB/dysfunctional voiding and patients with neurogenic pathologies. No major complications have been reported.

Randomized controlled trials are available only for OAB (4 studies) and CPP/PBS (2 studies). Level 1 evidence of PTNS efficacy for OAB is available. Promising results, to be confirmed by randomized controlled studies, have been obtained in the remaining indications considered.

**Conclusions:**

PTNS is an effective and safe option to treat OAB patients. Further studies are needed to assess the role of PTNS in the remaining indications and to evaluate the long term durability of the treatment. Further research is needed to address several unanswered questions about PTNS.

## Background

Percutaneous Tibial Nerve Stimulation (PTNS) is a lower urinary tract neuromodulation technique performed by percutaneous electrical stimulation of the posterior tibial nerve. This technique was described by Stoller in the late 1990s for the treatment of overactive bladder syndrome [1]. The needle insertion point, situated 4–5 cm cephalad to the medial malleolus, has previously been acknowledged as a neural access point for the regulation of bladder and pelvic floor function. Furthermore, experiments on animals demonstrated that the electrical stimulation of the hind leg produces detrusor inhibition [2]. Basing his research on these concepts, McGuire [3] showed that the transcutaneous electric stimulation of the posterior tibial nerve can suppress neurogenic detrusor overactivity.

### Description of the technique

**Figure 1:**
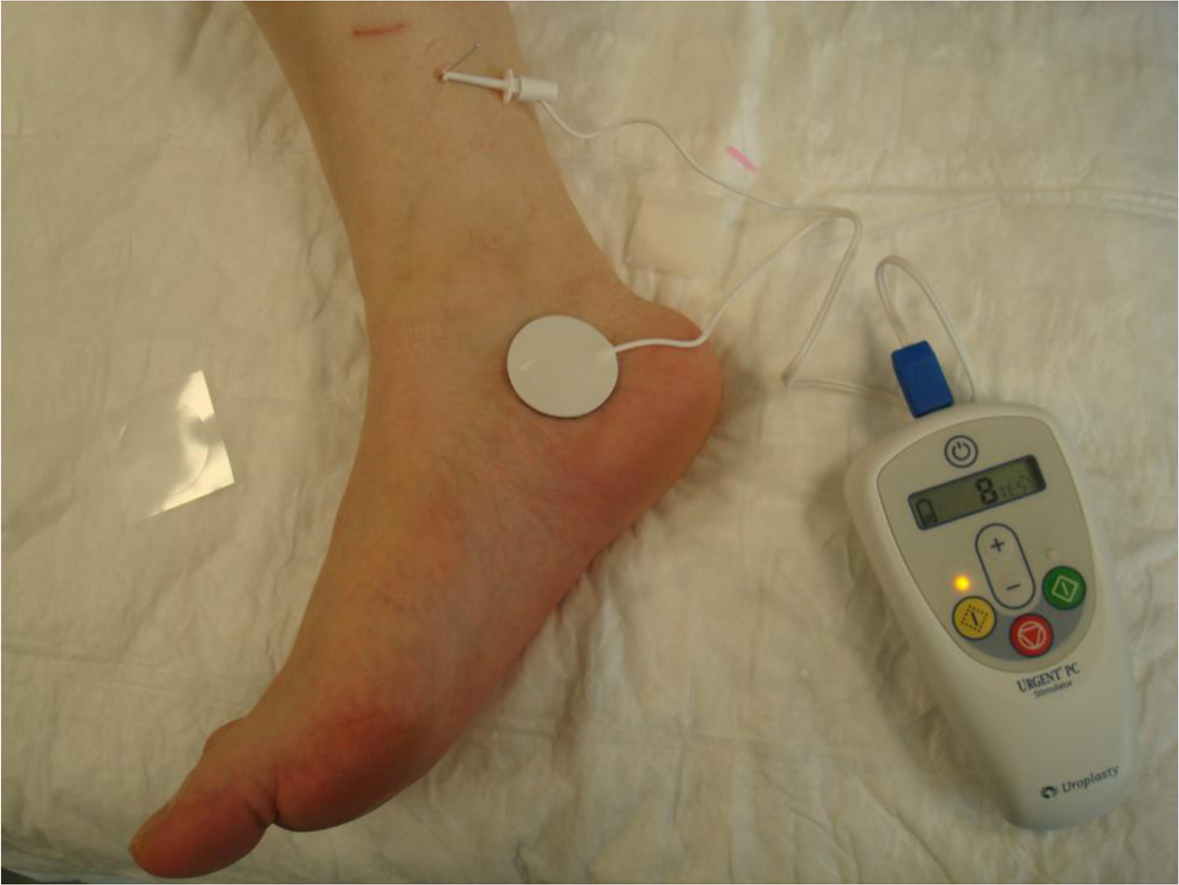
Stimulator and technique for percutaneous tibial nerve stimulation (PTNS).

The technique consists of stimulating the nerve by means of a 34 gauge needle electrode inserted 4–5 cm cephalad to the medial malleolus. Once the current is applied, the flexion of the big toe or the movement of the other toes confirms the correct positioning of the needle electrode. The electric current is a continuous, square wave form with a duration of 200 μs and a frequency of 20 Hz. The current intensity is determined by the highest level tolerated by the patient. In Figure 1 the stimulator (Urgent® PC, Uroplasty, Minnetonka, MN, USA) and the technique of stimulation are represented. The stimulation sessions last for 30 minutes and are performed once a week for 10–12 weeks in the majority of published papers. In a report published by Finazzi Agrò et al. [4], the possibility of a more frequent stimulation was analysed: stimulation performed 3 times a week obtained the same results obtained as a weekly stimulation protocol. The advantage of more frequent sessions is to obtain effects in 4 weeks instead of 12: results seemed to be dependent upon the number of stimulations performed and not the time elapsed from the beginning of the stimulation program [4]. In a recent study [5], a protocol of weekly PTNS sessions performed for 6 weeks was evaluated in women with overactive bladder syndrome. The Authors found that this shortened protocol obtained a positive response in 69,7% of 43 women.

#### Mechanisms of action

To date despite of its great clinical use, PTNS mechanism of action still remains unclear even though in the last years several studies have tried to better clarify it.

Some papers have shown that an effect of detrusor inhibition can be found after hind leg or pudendal electric stimulation in animal models [2,6]. In two very recent studies [2,7] Tai and co-workers have found that irritation induced bladder overactivity is suppressed by tibial nerve stimulation in cats. A 30 minute stimulation at both low (5 Hz) and high (30 Hz) frequencies was able to induce prolonged poststimulation inhibition of bladder activity, which lasted for more than 2 h and significantly increased bladder capacity.

Danisman [8] found that after PTNS the mast cells count in the bladder of female rats diminished.

Another study, as described by Chang and colleagues [9], shows that PTNS could produce effects on the (sacral) spinal cordby reducting C-fos expression (a marker of neuronal metabolic activity), in rat sacral spinal cord, after electrical stimulation of the hind leg.

An effect on supraspinal centers, has also been demonstrated in humans in a paper published by Finazzi Agro [10]. The Authors found a significant increase in amplitude of long latency somatosensory evoked potentials (LL-SSEP) recorded 24 hours after the end of a 12 session PTNS program. This finding could reflect a modification in elaboration mechanisms of sensory stimuli and it suggests a possible reorganization of cortical excitability after PTNS.

In conclusion, data available do not permit to draw definitive conclusions about PTNS mechanisms and sites of action; the results of this treatment can be due to effects on different areas of the central nervous system, but also to a peripheral effect on the target organ.

### Aim of study

Despite the lack of certainty about the mechanism of action of PTNS, in the last decade this technique has been widely used for the treatment of overactive bladder syndrome (OAB) and results of PTNS on non-obstructive urinary retention (NOUR), neurogenic bladder, paediatric voiding dysfunctions and chronic pelvic pain/painful bladder syndrome (CPP/PBS) have been described as well.

Aim of this systematic review, reported accordingly to the PRISMA statements [11], was to assess PTNS efficacy not only in OAB but also in other common urological conditions and to underline gaps in the present knowledge where research is still needed.

## Methods

### Eligibility criteria

All studies published on international peer reviewed journals have been considered. Only papers in English language were included in the review. Papers with only abstract were excluded. No publication date restriction was imposed. Participants of any age, sex, affected by any pathology of urological interest were considered. Only study describing effects of a percutaneous electrical stimulation of the posterior tibial nerve were considered. Primary outcome measure was the percentage of patients considered improved, independently by the definition of improvement used by the Authors. Several secondary outcome measures were considered.

### Information sources

A literature search using MEDLINE and ISI web was performed. The last literature search was run on December 2012. The search was conducted by two physicians independently.

### Search

Search terms used were “tibial nerve” combined with each of the following: “overactive bladder syndrome”, “urinary retention”, “neurogenic bladder”, “voiding dysfunction”, “chronic pelvic pain”, “painful bladder syndrome”, “Stoller afferent nerve stimulation”. Related articles of pertinent papers were also searched.

### Study selection

Eligibility assessment was performed independently by two reviewers who screened papers titles and abstracts. Case Reports were excluded.

### Data collection process

One review author extracted the following data from included studies and the second author checked the extracted data. Disagreements were resolved by discussion between the two review authors; if no agreement could be reached, it was planned a third author would decide.

### Data items

Information was extracted from each included study on: condition treated, type of study, mean or median age of patients population, percentage of female patients, definition of improvement, number and percentage of improved patients, treatment of control group (if present).

### Risk of bias across studies

An evaluation of level of evidence (based on the Oxford Centre for Evidence-Based Medicine criteria [12] of PTNS efficacy) was performed for each paper.

**Figure 2:**
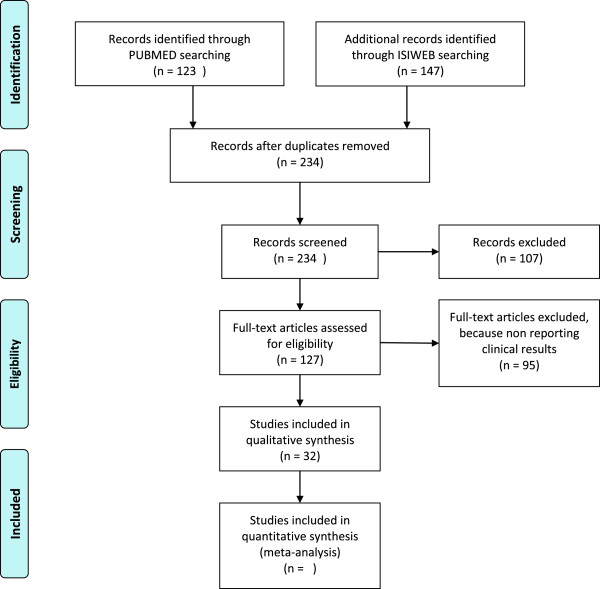
PRISMA Flow diagram showing the search strategy used to identify studies to include in the review.

Our search strategy is shown in Figure 2.

## Results

### Evidence synthesis

We included 32 studies, with a total of 1087 adult participants between 18 and 82 years of age and 62 children between 1 and 17 years old. There were 6 Randomized clinical trial (RCT) studies: two compared PTNS with Sham studies, two compared PTNS with anticholinergic therapy, one compared PTNS with placebo therapy and one with ‘no treatment’ group.

There are 16 studies about OAB, 4 studies about NOUR, 9 studies about CPP/PBS, 3 studies about neurogenic bladder. Four studies are in children (1 with OAB, 1 OAB and NOUR and a OAB and Dysfunctional voiding). Only 6 studies are Randomized clinical trials: 4 in the OAB group and 2 in the CPP/PBS group. In the OAB RCT studies, control groups are different: Tolterodine, placebo, sham, oxybutynin. In the CPP/PBS group one RCT study uses sham and one has ‘no treatment’. There are 9 multicenter studies.

The inclusion criteria were:

– Participants: patients (adults and children) with idiopathic OAB, NOUR, CPP/PBS and neurogenic bladder;

– Intervention: Percutaneous tibial nerve stimulation (PTNS);

– Comparison: Sham nerve stimulation; anticholinergic medications; placebo;

– Outcome measures: cure/improvement in symptoms at the end of initial treatment program.

The exclusion criteria were:

– Papers with only abstract;

– Non-English Articles;

– Case reports;

– Papers about PTNS non reporting clinical results.

### Participants and intervention

OAB studies involved a total of 640 treated patients and 189 control patients, NOUR studies involved 81 treated patients, CPP/PBS involved 233 treated patients and 56 control patients. Children studies are on 62 patients and Neurogenic bladder studies are on 72 patients.

In all studies the rate of female patients is higher than the rate of male patients. Two studies are only on female. In five studies sex is not specified. In adult studies mean age ranges between 45 and 65 years old.

### Comparison

Two studies compared PTNS with Sham nerve stimulation, two studies compared PTNS with anticholinergic medications, one with placebo and one with ‘no treatment’ group.

### Outcomes

Six studies evaluate only urodynamic outcome, 20 only clinical outcome and 6 urodynamic and clinical outcome.

#### Results in overactive bladder syndrome

Several studies have been published evaluating the effects of PTNS on OAB [13-19]. According to these studies, the overall percentage of patients classified as “successfully treated” was 54.5-79.5%. Of note, the definition of “success” differs among studies from the use of urodynamic data to clinical parameters and quality of life measures. In spite of these differences, the reported success rates are of clinical interest, especially because many were obtained from a population of patients who were already non responsive to conventional therapies. Improvements are reported not only in symptoms, but also in urodynamic observations. Klingler [13] reported a reduction of detrusor overactivity and Vandoninck showed an increase of the cystometric capacity and of the threshold of appearance of involuntary detrusor contractions [17].

##### Randomized controlled trials on OAB

More recently, some randomized controlled studies on PTNS as treatment of OAB have been published. Peters et al. [20] provided the results of a randomized controlled study comparing PTNS to tolterodine 4 mg extended release. The subjects’ global response assessments of overactive bladder symptoms was improved from baseline in a significantly higher percentage of patients in the PTNS arm compared to the tolterodine arm (79.5% reporting cure or improvement vs. 54.8%, p = 0.01). Objective measures (reductions in urinary frequency, incontinence episodes, urge severity and night-time voids; improvement in voided volume) improved similarly in both groups. The Authors state that PTNS is safe and offers improvements of OAB symptoms, with objective effectiveness comparable to pharmacotherapy.

After first developing a validated sham for PTNS [21], Peters randomized a total of 220 adults with OAB to PTNS or sham therapy [22]. PTNS subjects achieved statistically significant improvement in bladder symptoms with 54.5% reporting moderately or markedly improved responses compared to 20.9% of sham subjects from baseline (p < 0.001). According to Authors, PTNS therapy is safe and effective in treating OAB and superior to a placebo.

More recently, Finazzi Agrò [23] provided a prospective double blind, placebo controlled study aimed to investigate the possible placebo effects of PTNS on detrusor overactivity incontinence. Patients were randomly assigned either to PTNS or to placebo group. Patients showing a reduction >50% of urge incontinence episodes were considered "responders". A statistically significant difference between responders’ rates was found (71% in PTNS group vs 0% in placebo group, p < 0.0001). Improvement in the number of incontinence episodes, number of voids, voided volume and Incontinence quality of life (I-QoL) score were statistically significant in the PTNS group but not in placebo group. The authors concluded that the relevance of a placebo effect was negligible in this patients' population.

Results for OAB are reported in Table 1: positive data on the efficacy of PTNS in this condition are reported by all Authors. Three randomized controlled trials (level of evidence 1) are available for PTNS as treatment for this condition.

**Table 1:** Results of the use of PTNS in OAB Patients

**Authors**	**Years**	**Control group**	**PTNS**			**Other**				**RCT**	**Methods**	**Level**	**Results**	**Female n(%)**		**Mean age**		**Multicenter**
			**n**	**Postive**	**%**	**n**	**Postive**	**%**	**R**					**PTNS**	**Control group**	**PTNS group**	**Control group**	
Klingler HC	2000 [13]	N/A	15	10	67					N	Urodynamic and clinical	3	<10 voids/day <2voids/night PadTest (g) 10	73		N/A		N
Govier FE	2001 [14]	N/A	47		71					N	Urodynamic	2-3	25% reduction in mean daytime voiding frequency	90		57.4 (24–80)		Y
van Balken MR	2001 [15]	N/A	37	22	59					N	Clinical	2-3	Request for continued chronice treatment	73		52.5 (23–74)		Y
Vandonink V	2003 [16]	N/A	35	24	69					N	Clinical	2-3	Continuing treatment	71		57 (29–82)		Y
Vandonink V	2003 [17]	N/A	60	34	57					N	Urodynamic	2-3	50% reduction					Y
Peters KM	2009 [20]	Tolterodine	44	35	80	42	23	55	0.01	Y	Clinical	2-3	Improvemtn for cure in 79.5% compared to 54.8% in the tolterodine arm	96	92	57.5	58.2	Y
Peters KM	2010 [22]	Sham	110	60	55	110	23	21	<0.001	Y	Clinical	1	Improvement in overall bladder symptoms	78	80	62.5	60.2	N
Finazzi-Agro E	2010 [23]	Placebo	17	12	71	15	0	0	<0.001	Y	Clinical	1	50% reduction	100	100	44.9	45.5	N
Amarenco G	2003 [38]	N/A	44	22	50					N	Clinical	1		66		53.3		N
van Der pal F	2005 [26]	N/A	11	11	100					N	Clinical		Improvement in overall bladder symptoms	54.5		51 (33–66)		N
Karademir K	2005 [18]	Oxybutynin + PTNS	21	13	62	22	18	82	<0.0001	Y	Clinical		Improvement in overall bladder symptoms					Y
de Séze M	2011 [45]	N/A	70	58	82.8					N	Urodynamic and clinical		Improvement in 82.6% and 83.3% of the patients on day 30 and day 90 regarding symptoms and QoL					N
van Balken MR	2006 [44,47]	N/A	132	43 O/68 S	32,6 O/51,5 S					N	Clinical		Improvement QoL	61.3		53 (21–82)		N
Nuhoglu B	2005 [19]	N/A	35	19	54					N	Urodynamic and clinical		Improvement Urgency and QoL	100		47.3 (35–57)		

#### Results in non-obstructive urinary retention

PTNS has been used also in the treatment of non-obstructive urinary retention (NOUR) and the experience in this field is limited to few published papers. According to these papers, the percentage of patients successfully treated is good, varying from 41 to 100%, according to the parameters chosen to classify “success” [13,24,25]. In a study published by Vandoninck [24], the primary outcome measure was a reduction of the total catheterized volume per 24 hours. Using a reduction of >50%, the percentage of responders was 41%; using a reduction rate of >25%, the percentage of responders was 67%.

According to another paper from the same authors [25], an improvement of the urodynamic parameters of the voiding phase (maximum flow, detrusor pressure at maximum flow, post-void residual urine) was also observed.

Van der Pal [26] showed that PTNS has an effect in QoL of all patients investigated and a reduction of at least two pads/day recorded in the bladder diary.

Results for NOUR are reported in Table 2: positive data on the efficacy of PTNS in this condition are reported by all Authors. No randomized controlled trial is available for PTNS as treatment for this condition; only prospective non randomized trials are available (level of evidence 2–3).

**Table 2:** Results of PTNS in Patients affected by non obstructive urinary retention

**Authors**	**Years**	**Control group**	**PNT**			**RCT**	**Methods**	**Level**	**Results**	**Female (%)**	**Mean age**	**Multicenter**
			**n**	**Positive**	**%**							
van Balken MR	2001 [15]	N/A	12	7	58	N	Clinical	2-3	Request for continued chronice treatment	58	58, 8	N
Vandoninck V	2003 [16,17]	N/A	39	16	41	N	Clinical	2-3	50% reduction of catherterized volume	69	53 (28–77)	Y
Vandoninck V	2004 [24]	N/A	39	16	41	N	Urodynamic	3	50% reduction of catherterized volume	69	53 (28–77)	Y
van der pal F	2006 [26]	N/A	30	29	100	N	Bladder Diary and QoL	2-3	Improvement BD and QoL	86.6	51 (20–72)	N

#### Results in chronic pelvic pain/painful bladder syndrome

Few studies have evaluated the effect of PTNS on CPP/PBS [27-32]. According to Van Balken [27], this technique seems to be effective in as much as 42% of patients with CPP. In the same group of patients, Kim [28] found that 90% showed an improvement >25% in the VAS score for pain, with 60% reaching improvement >50%. Kabay [29] evaluated the efficacy of PTNS in the treatment of patients with category IIIB chronic non-bacterial prostatitis. 89 patients were randomized to receive either PTNS (n = 45) or sham treatment (n = 44). A complete response on pain and symptoms was observed after PTNS in 40% and 66.6% of the patients, whereas a partial response was observed in 60% and 33.3% of the patients, respectively; no significant results were seen after sham treatment.

Two studies from Zhao [30,31] evaluated PTNS for the treatment of PBS/interstitial cystitis (IC). In the first one [30], after a prospective evaluation in 14 patients with refractory IC, the authors concluded that PTNS had no significant clinical effect over 10 weeks of treatment. In a more recent study [31], the same author evaluated the efficacy of PTNS performed twice a week in 18 female patients with IC. 44.4% of patients evaluated the trial effective and showed a significant improvement in bladder capacity. Baykal [32] evaluated the effect of intravesical heparin and PTNS in 10 subjects with IC. After 2 and 12 months of treatment, patients showed significant reduction of symptoms and of Wisconsin pain scores as well as an increase of cystometric capacity. The authors concluded that the combination of intravesical heparin and peripheral neuromodulation seems to be an alternative for patients with IC who were not responsive to other treatments.

Congregado [33] described a significant improvement in all lower urinary tract irritative symptoms of 51 female patients studied. Gokyldiz [34] reported a 100% of clinical success in 12 patients with chronic pelvic pain treated with PTNS.

Results for CPP/PBS are reported in Table 3: positive data on the efficacy of PTNS in this condition are reported by all but one Authors. Only one randomized controlled trial is available for PTNS as treatment for CPP (level 1, one paper); several prospective non randomized trials are available (level of evidence 3).

**Table 3:** Efficacy of PTNS in chronic pelvic pain/painful bladder syndrome

**Authors**	**Years**	**Control group**	**PNT**			**Other**			**RCT**	**Methods**	**Level**	**Results**	**Female (%)**		**Mean age (range)**		**Multicenter**
			**n**	**Positive**	**%**	**n**	**Positive**	**%**					**PNT group**	**Control group**	**PNT group**	**Control group**	
van Balken MR	2003 [27]	N/A	33	14	42				N	Clinical	3	Mean VAS for pain	33		51.6 (25–79)		N
Kim SW	2007 [28]	N/A	15	9	60				N	Clinical	3	VAS score for pain reduction >50%			60 (41–78)		N
Kabay S	2009 [29]	Sham	45	18	40	44	0	0	Y	Clinical	1	VAS score for pain reduction >50%	0	0	37.9 (range 24–51)	38.5 (range 25)	N
Zhao J	2004 [31]	N/A	14	0	0				N	Clinical	3	VAS Scale reduction	93				N
Zhao J	2008 [31]	N/A	18	8	44				N	Clinical	3	Bladder capacity increases	100		60		N
Baykal K	2005 [32]	N/A	10	10	100				N	Clinical	3	Wiscosin pain score	80		49 (40–62)		N
Congregado Ruiz B	2004 [33]	N/A	51	51	100				N	Clinical		Improvement QoL	100		55 (18–74)		N
Gokyildiz S	2012 [34]	No treatment	12	12	100	12	0	0	Y	Clinical		Improvement QoL	100	100	/	/	N

#### Results in children

PTNS seems to be effective in the treatment of non-neurogenic lower urinary tract dysfunctions of children: 60-80% of children with OAB and 43-71% of children with urinary retention showed a significant improvement [35-37]. De Gennaro [36] found that PTNS is generally well accepted by children, with low scores of a visual analog scale for pain, that further decreased during the treatment. Efficacy at 2 year follow up was maintained [37].

Results in children are reported in Table 4: no randomized controlled trial is available for PTNS as treatment for dysfunctional voiding/OAB in children; only prospective non randomized trials are available (level of evidence 2–3).

**Table 4:** Efficacy of PTNS use in children

**Authors**	**Years**	**Control group**	**PNT**			**RCT**	**Methods**	**Level**	**Results**	**Female (%)**	**Mean age**	**Multicenter**
			**n**	**Positive**	**%**							
Hoebeke P	2002 [35]	N/A	31	27	87		Clinical and Urodynamic findings	2-3	Clinical and shape of the uroflowmetry curve	48	11, 7	N
De Gennaro M	2004 [36]	N/A	10	8	80	N		2-3	Clinical and Urodynamic findings	60	9	N
De Gennaro M	2004 [36]	N/A	7	5	71	N		2-3	Clinical and Urodynamic findings	57	12	N
Capitanucci ML	2009 [37]	N/A	14	12	86	N	Clinical and Urodynamic findings	2-3	Clinical findings	NS	NS	N
Capitanucci ML	2009 [37]	N/A	14	14	100	N		2-3	Clinical findings	NS	NS	N

#### Results in patients with neurogenic bladder

Few reports have been published on the effects of PTNS in patients with neurogenic bladder. Acute urodynamic effect of PTNS were observed in a mixed population of OAB patients, most of whom neurologically impaired (multiple sclerosis –MS-, spinal cord injury -SCI-, Parkinson’s disease -PD-). During stimulation, an increase of first involuntary detrusor contraction volume and of cystometric capacity was found [38]. Similar results were observed by Kabay [39] in PD patients with detrusor overactivity. On the other hand, Fjorback [40] failed to obtain acute urodynamic reductions of detrusor overactivity in MS patients.

Kabay and Gobbi [41,42] investigated the effect of PTNS on the lower urinary tract symptoms in MS patients with detrusor overactivity and lower urinary tract symptoms (LUTS), respectively. After 12 weeks, statistically significant improvements in several urodynamic and clinical parameters were observed. Both authors concluded that PTNS is effective to improve LUTS in MS patients.

Results in neuropathic bladder are reported in Table 5: positive data on the efficacy of PTNS in this condition are reported by all Authors. No randomized controlled trial is available for PTNS as treatment for this condition; only prospective non randomized trials are available (level of evidence 2–3).

**Table 5:** Results of the use of PTNS in Patients affected by neurogenic bladder

**Authors**	**Years**	**Control group**	**PNT**			**RCT**	**Methods**	**Level**	**Results**	**Female (%)**	**Mean age**	**Multicenter**
			**n**	**Positive**	**%**							
Kabay S	2009 [29,39,41]	N/A	32	15	47	N	Urodynamic	2-3	50% improvement cystometric capacity	41	64 (44–78)	N
Gobbi C	2011 [42]	N/A	21	16	76	N	Clinical	2-3	Patient perception of Bladder Cond	76	46 (29–62)	N
Kabay S	2009 [29,39,41]	N/A	19	19	100	N	Urodynamic		Custometry parameters	100		N

#### Complications

No major complications are reported in literature, following PTNS treatment. Only mild to moderate pain in the site of the puncture was reported by some authors; the majority of patients, with the inclusion of children [35-37], seem to tolerate perfectly the positioning of the needle and the subsequent stimulation.

#### Long term durability

The only available long term study on results of PTNS on the treatment of overactive bladder was published by MacDiarmid et al in 2010 [43]. Subject global response assessments showed sustained improvement from 12 weeks at 6 and 12 months, with 94% and 96% of responders, respectively.

Patients in this trial were receiving periodic PTNS sessions. Van der Pal [44] found that, 6 weeks after initial PTNS therapy, 64% of patients showed a worsening of symptoms, thus underlining the need of a maintenance stimulation protocol. The need for repeated stimulation sessions could be less common in children: according to Capitanucci, maintenance stimulation sessions are needed only in 29% of children with dysfunctional voiding and in 50% of children with overactive bladder [37].

The need of repeated stimulation sessions, possibly for long time or lifelong, is probably the major limit of PTNS, requiring either periodic office based procedures, or a home based treatment. Transcutaneous stimulation, as proposed by McGuire [3], could be an alternative for chronic treatment. Some very recent papers evaluated the efficacy of transcutaneous tibial nerve stimulation (TTNS) in the treatment of OAB in multiple sclerosis patients [45] and of urgency incontinence in older women [46].

## Discussion

### Recommendations for further research

#### Efficacy of PTNS

Level 1 evidence is produced by few studies for efficacy of PTNS in the treatment of OAB/urge incontinence. PTNS seems to be an efficacious and safe treatment for OAB that could be highly recommended. Nevertheless, this evidence needs to be confirmed by further good quality randomized controlled studies and meta-analysis of them.

For all the remaining indications considered in this systematic review only 2–3 level of evidence of efficacy is available for PTNS (only one RCT for CPP/PBS is available). RCT for PTNS in these indications are highly recommended.

#### Stimulation protocol

Little is known about the effects of the electric stimulation parameters and the stimulation protocols on PTNS efficacy. Further studies are needed to identify the best electric parameters and the best protocols for every indications as well as possible effects of a combination therapy with drugs (e.g. antimuscarinics for OAB or intravesical glycosaminoglycans for painful bladder syndrome).

#### Safety

According to published data, PTNS is safe and well tolerated. Nevertheless, future studies will have to include safety data of the technique.

#### Predictive factors of PTNS success

Very few data on predictive factors of success of PTNS are available.

The urodynamic characteristics of OAB patients seem to be relevant: OAB patients not showing detrusor overactivity (or showing it only at higher bladder volumes) seem to be more prone to respond to treatment [17]. In NOUR, patients with milder symptoms seem to respond better [25]. Bad mental health (as measured with the SF-36 Mental Component Summary) seems to be a negative predictive factor for success of PTNS in patients with OAB, NOUR or CPP/PBS [47].

Studies on subgroups of patients in the different indications considered are needed, to find patients more prone to respond to this treatment, with the aim to reduce the number of patients unsuccessfully treated, thus reducing the costs.

#### Long term durability

Only one long term (12 months) study on results of PTNS on the treatment of overactive bladder is available [43]. No long term studies are available for the remaining indications. Further long term studies are needed.

As already mentioned, the need of repeated stimulation sessions is an important drawback of PTNS, making this technique time consuming for the patients and the health professionals. Further studies on alternative possible treatments (e.g. home based transcutaneous stimulation) are needed.

#### Mechanisms of action

Few data are available about possible mechanisms of action of PTNS. Studies on animal models and on humans, possibly using central nervous system functional imaging techniques are to be encouraged.

#### Economic data

Analysis of the costs of PTNS both in the short and in the long term, in relation with the patients’ quality of life improvement, would be very useful to understand the cost-effectiveness of this treatment.

## Conclusions

PTNS is an effective treatment for patients with OAB syndrome non responding to conservative therapies. Results from randomized controlled studies demonstrate that the success rate of PTNS is statistically superior to that of placebo. The durability of the improvement obtained by PTNS has also been demonstrated with periodic stimulations to sustain the therapeutic effects. Finally PTNS is safe, with no major complications reported in literature. In consideration, of these potentialities, as suggested by some authors [48], PTNS could be offered early in the course of OAB treatment.

Promising results, to be confirmed by randomized controlled studies, have been obtained in non-obstructive urinary retention, CPP/PBS and urinary disorders in children. Further studies are needed to assess the exact role of PTNS in these indications and to evaluate the long term durability of the treatment. Further research is needed as well to assess several still unanswered questions about PTNS.

## Abbreviations

PTNS: Percutaneous tibial nerve stimulation; OAB: Overactive bladder syndrome; NOUR: Non obstructive urinary retention; CPP/PBS: Chronic pelvic pain/painful bladder syndrome; RCT: Randomized clinical trial; I-QoL score: Incontinence quality of life score; IC: Interstitial cystitis; MS: Multiple sclerosis; SCI: Spinal cord injury; PD: Parkinson’s disease; LUTS: Lower urinary tract symptoms; TTNS: Transcutaneous tibial nerve stimulation.

## Competing interests

The authors declare that they have no competing interests.

## Authors' contributions

GG and VI have made substantial contributions to acquisition, analysis and interpretation of data. EFA, ADS and AA have made substantial contributions to conception and design of the study. LT has been involved in drafting the manuscript. CDN and EFA have been involved in revising the manuscript critically for important intellectual content. All authors read and approved the final manuscript.

## Pre-publication history

The pre-publication history for this paper can be accessed here:

http://www.biomedcentral.com/1471-2490/13/61/prepub

## References

[B1] Stoller ML Afferent nerve stimulation for pelvic floor dysfunction Eur Urol 1999 35 Suppl 2 132

[B2] Tai C Chen M Shen B Irritation induced bladder overactivity is suppressed by tibial nerve stimulation in cats J Urol 2011 186 1 326 330 10.1016/j.juro.2011.04.023 21600604 PMC3138204

[B3] McGuire EJ Zhang SC Horwinski ER Treatment of motor and sensory detrusor instability by electrical stimulation J Urol 1983 129 1 78 79 6600794 10.1016/s0022-5347(17)51928-x

[B4] Finazzi Agrò E Campagna A Sciobica F Posterior tibial nerve stimulation: is the once-a-week protocol the best option? Minerva Urol Nefrol 2005 57 2 119 123 15951736

[B5] Yoong W Ridout AE Damodaram M Neuromodulative treatment with percutaneous tibial nerve stimulation for intractable detrusor instability: outcomes following a shortened 6-week protocol BJU Int 2010 106 11 1673 1676 10.1111/j.1464-410X.2010.09461.x 20590544

[B6] Jiang CH Lindstrom S Prolonged enhancement of the micturition reflex in the cat by repetitive stimulation of bladder afferents J Physiol 1999 517 Pt 2 599 605 10332105 10.1111/j.1469-7793.1999.0599t.x PMC2269345

[B7] Tai C Shen B Chen M Prolonged poststimulation inhibition of bladder activity induced by tibial nerve stimulation in cats Am J Physiol Renal Physiol 2011 300 2 385 392 10.1152/ajprenal.00526.2010 PMC3044011 21106856

[B8] Danisman A Kutlu O Akkaya E Tibial nerve stimulation diminishes mast cell infiltration in the bladder wall induced by interstitial cystitis urine Scand J Urol Nephrol 2007 41 2 98 102 10.1080/00365590600911233 17454946

[B9] Chang CJ Huang ST Hsu K Electroacupuncture decreases c-fos expression in the spinal cord induced by noxious stimulation of the rat bladder J Urol 1998 160 6 Pt 1 2274 2279 9817383 10.1097/00005392-199812010-00099

[B10] Finazzi Agrò E Rocchi C Pachatz C Percutaneous tibial nerve stimulation produces effects on brain activity: study on the modifications of the long latency somatosensory evoked potentials Neurourol Urodyn 2009 28 4 320 324 10.1002/nau.20651 19090588

[B11] Moher D Liberati A Tetzlaff J Altman DG The PRISMA Group (2009): Preferred Reporting Items for Systematic Reviews and Meta-Analyses: The PRISMA Statement Open Med 2009 3 3 123 130 PMC3090117 21603045

[B12] OCEBM Table of Evidence Working Group "The Oxford 2011 Table of Evidence". Oxford Centre for Evidence-Based Medicinehttp://www.cebm.net/index.aspx?o=5653

[B13] Klingler HC Pycha A Schmidbauer J Use of peripheral neuromodulation of the S3 region for treatment of detrusor overactivity: a urodynamic-based study Urology 2000 56 5 766 771 10.1016/S0090-4295(00)00727-5 11068296

[B14] Govier FE Litwiller S Nitti V Percutaneous afferent neuromodulation for the refractory overactive bladder: results of a multicenter study J Urol 2001 165 4 1193 1198 10.1016/S0022-5347(05)66469-5 11257669

[B15] van Balken MR Vandoninck V Gisolf KW Posterior tibial nerve stimulation as neuromodulative treatment of lower urinary tract dysfunction J Urol 2001 166 3 914 918 10.1016/S0022-5347(05)65863-6 11490245

[B16] Vandoninck V Van Balken MR Finazzi Agrò E Posterior tibial nerve stimulation in the treatment of urge incontinence Neurourol Urodyn 2003 22 1 17 23 10.1002/nau.10036 12478596

[B17] Vandoninck V van Balken MR Finazzi Agrò E Percutaneous tibial nerve stimulation in the treatment of overactive bladder: urodynamic data Neurourol Urodyn 2003 22 3 227 232 10.1002/nau.10111 12707873

[B18] Karademir K Baykal K Sen B A peripheric neuromodulation technique for curing detrusor overactivity: Stoller afferent neurostimulation Scand J Urol Nephrol 2005 39 3 230 233 10.1080/00365590510031147 16118096

[B19] Nuhoğlu B Fidan V Ayyildiz A Stoller afferent nerve stimulation in woman with therapy resistant over active bladder, a 1-year follow up Int Urogynecol J Pelvic Floor Dysfunct 2006 17 3 204 207 10.1007/s00192-005-1370-x 16049624

[B20] Peters KM MacDiarmid SA Wooldridge LS Randomized trial of percutaneous tibial nerve stimulation versus extended-release tolterodine: results from the overactive bladder innovative therapy trial J Urol 2009 182 3 1055 1061 10.1016/j.juro.2009.05.045 19616802

[B21] Peters K Carrico D Burks F Validation of a sham for percutaneous tibial nerve stimulation (PTNS) Neurourol Urodyn 2009 28 1 58 61 10.1002/nau.20585 18671297

[B22] Peters KM Carrico DJ Perez-Marrero RA Randomized Trial of Percutaneous Tibial Nerve Stimulation Versus Sham Efficacy in the Treatment of Overactive Bladder Syndrome: Results From the SUmiT Trial J Urol 2010 183 4 1438 1443 10.1016/j.juro.2009.12.036 20171677

[B23] Finazzi-Agro E Petta F Sciobica F Percutaneous Tibial Nerve Stimulation effects on detrusor overactivity incontinence are not due to a placebo effect: a randomized double-blind placebo-controlled trial J Urol 2010 184 5 2001 2006 10.1016/j.juro.2010.06.113 20850833

[B24] Vandoninck V van Balken MR Finazzi Agrò E Posterior tibial nerve stimulation in the treatment of idiopathic nonobstructive voiding dysfunction Urology 2003 61 3 567 572 10.1016/S0090-4295(02)02378-6 12639649

[B25] Vandoninck V Van Balken MR Finazzi Agrò E Posterior tibial nerve stimulation in the treatment of voiding dysfunction: Urodynamic data Neurourol Urodyn 2004 23 3 246 251 10.1002/nau.10158 15098221

[B26] van der Pal F van Balken MR Heesakkers JP Correlation between quality of life and voiding variables in patients treated with percutaneous tibial nerve stimulation BJU Int 2006 97 1 113 116 10.1111/j.1464-410X.2006.05860.x 16336339

[B27] van Balken MR Vandoninck V Messelink BJ Percutaneous tibial nerve stimulation as neuromodulative treatment of chronic pelvic pain Eur Urol 2003 43 2 158 163 10.1016/S0302-2838(02)00552-3 12565774

[B28] Kim SW Paick JS Ku JH Percutaneous posterior tibial nerve stimulation in patients with chronic pelvic pain: a preliminary study Urol Int 2007 78 1 58 62 10.1159/000096936 17192734

[B29] Kabay S Kabay SC Yucel M Efficiency of posterior tibial nerve stimulation in category IIIB chronic prostatitis/chronic pelvic pain: a Sham-Controlled Comparative Study Urol Int 2009 83 1 33 38 10.1159/000224865 19641356

[B30] Zhao J Nordling J Posterior tibial nerve stimulation in patients with intractable interstitial cystitis BJU Int 2004 94 1 101 104 10.1111/j.1464-410X.2004.04909.x 15217440

[B31] Zhao J Bai J Zhou Y Posterior tibial nerve stimulation twice a week in patients with interstitial cystitis Urology 2008 71 6 1080 1084 10.1016/j.urology.2008.01.018 18372023

[B32] Baykal K Senkul T Sen B Intravesical heparin and peripheral neuromodulation on interstitial cystitis Urol Int 2005 74 4 361 364 10.1159/000084439 15897705

[B33] Congregado Ruiz B Pena Outeiriño XM Campoy Martínez P León Dueñas E Leal López A Peripheral afferent nerve stimulation for treatment of lower urinary tract irritative symptoms Eur Urol 2004 45 65 69 10.1016/j.eururo.2003.08.012 14667518

[B34] Gokyldiz S Effects of percutaneous tibial nerve stimulation therapy on chronic pelvic pain Gynecol Obstet Invest 2012 73 2 99 105 Epub 2012 Jan 20 10.1159/000328447 22269443

[B35] Hoebeke P Renson C Petillon L Percutaneous electrical nerve stimulation in children with therapy resistant nonneuropathic bladder sphincter dysfunction: a pilot study J Urol 2002 168 6 2605 2607 10.1016/S0022-5347(05)64227-9 12441995

[B36] De Gennaro M Capitanucci ML Mastracci P Percutaneous tibial nerve neuromodulation is well tolerated in children and effective for treating refractory vesical dysfunction J Urol 2004 171 5 1911 1913 10.1097/01.ju.0000119961.58222.86 15076308

[B37] Capitanucci ML Camanni D Demelas F Long-term efficacy of percutaneous tibial nerve stimulation for different types of lower urinary tract dysfunction in children J Urol 2009 182 Suppl 4 2056 2061 19695611 10.1016/j.juro.2009.03.007

[B38] Amarenco G Ismael SS Even-Schneider A Urodynamic effect of acute transcutaneous posterior tibial nerve stimulation in overactive bladder J Urol 2003 169 6 2210 2215 10.1097/01.ju.0000067446.17576.bd 12771752

[B39] Kabay SC Kabay S Yucel M Acute urodynamic effects of percutaneous posterior tibial nerve stimulation on neurogenic detrusor overactivity in patients with Parkinson's disease Neurourol Urodyn 2009 28 1 62 67 10.1002/nau.20593 18837432

[B40] Fjorback MV van Rey FS van der Pal F Acute urodynamic effects of posterior tibial nerve stimulation on neurogenic detrusor overactivity in patients with MS Eur Urol 2007 51 2 464 470 10.1016/j.eururo.2006.07.024 16956713

[B41] Kabay S Kabay SC Yucel M The clinical and urodynamic results of a 3-month percutaneous posterior tibial nerve stimulation treatment in patients with multiple sclerosis-related neurogenic bladder dysfunction Neurourol Urodyn 2009 28 8 964 968 10.1002/nau.20733 19373898

[B42] Gobbi C Digesu G Khullar V El Neil S Caccia G Zecca C Percutaneous posterior tibial nerve stimulation as an effective treatment of refractory lower urinary tract symptoms in patients with multiple sclerosis: preliminary data from a multicentre, prospective, open label trial Mult Scler 2011 17 12 1514 1519 Epub 2011 Jul 14 10.1177/1352458511414040 21757534

[B43] MacDiarmid SA Peters KM Shobeiri SA Long-term durability of percutaneous tibial nerve stimulation for the treatment of overactive bladder J Urol 2010 183 1 234 240 10.1016/j.juro.2009.08.160 19913821

[B44] van der Pal F van Balken MR Heesakkers JP Percutaneous tibial nerve stimulation in the treatment of refractory overactive bladder syndrome: is maintenance treatment necessary? BJU Int 2006 97 3 547 550 10.1111/j.1464-410X.2006.06055.x 16469023

[B45] de Sèze M Raibaut P Gallien P Transcutaneous posterior tibial nerve stimulation for treatment of the overactive bladder syndrome in multiple sclerosis: results of a multicenter prospective study Neurourol Urodyn 2011 30 3 306 311 10.1002/nau.20958 21305588

[B46] Schreiner L dos Santos TG Knorst MR Randomized trial of transcutaneous tibial nerve stimulation to treat urge urinary incontinence in older women Int Urogynecol J Pelvic Floor Dysfunct 2010 21 9 1065 1070 10.1007/s00192-010-1165-6 20458465

[B47] van Balken MR Vergunst H Bemelmans BL Prognostic factors for successful percutaneous tibial nerve stimulation Eur Urol 2006 49 2 360 365 10.1016/j.eururo.2005.10.019 16359781

[B48] Burks FN Peters KM Neuromodulation versus medication for overactive bladder: the case for early intervention Curr Urol Rep 2009 10 5 342 346 10.1007/s11934-009-0054-3 19709480

